# Integrated e-care for elderly mental reinforcement and social interaction: a pilot study (European project Sociable)

**Published:** 2012-09-04

**Authors:** P Mattarelli, S Passuti, M Fabbri, G Cannolicchio, S.P.A Maggioli

**Affiliations:** Cedaf Division, Italia; Cedaf Division, Italia; Cedaf Division, Italia; Cedaf Division, Italia; Cedaf Division, Italia

**Keywords:** ICT, MCI, Alzheimer disease, cognitive training, integrated care, social activation

## Abstract

**Purpose:**

Interest is growing in integrated systems of e-care for the frail elderly. The present project promotes cognitive training and assessment using ICT tools, stimulates the socialization, shares and monitors the situation of the elderly by different professionals, care givers and relatives.

**Context:**

Within the frame of the European Commission funded SOCIABLE Project, 348 elderly are participating in the trial.

**Data sources:**

The approach was pilot in seven different sites, across Italy (2 Geriatrics Units and 1 Municipality), Greece (1 Memory Clinic and 1 Municipality), Spain (1 University) and Norway (1 Municipality).

**Case description:**

SOCIABLE pilot study is a multi-national, multicenter, non-randomized, placebo-controlled efficacy study. Its primary objective is to evaluate the effects of a computer-based cognitive training and social activation program on the cognition, the affection and the functional abilities of cognitively intact elderly, patients with MCI and patients with mild AD. Conclusions: Two types of models of integrated care delivery for the frail elderly have been tested; one was characterized by group sessions in the pilot center assisted by caregiver or psychologist, another was stipulated in the use of ICT tools by the elderly at home. The first results of the trial will be illustrated.

**Discussion:**

Integrated care delivery can be achieved in various ways. Irrespective of which model is adopted were detected high levels of satisfaction and performance by all groups and an important ability to monitor results by medical experts and care givers.

